# AP4 is required for mitogen- and c-MYC-induced cell cycle progression

**DOI:** 10.18632/oncotarget.2348

**Published:** 2014-08-19

**Authors:** Rene Jackstadt, Heiko Hermeking

**Affiliations:** ^1^ Experimental and Molecular Pathology, Institute of Pathology, Ludwig-Maximilians-Universität München, Thalkirchner Str. 36, D-80337 Munich, Germany; ^2^ German Cancer Consortium (DKTK), D-69120 Heidelberg, Germany; ^3^ German Cancer Research Center (DKFZ), D-69120 Heidelberg, Germany

**Keywords:** AP4, AP-4, TFAP4, c-MYC, cell cycle, cytokinesis

## Abstract

AP4 represents a c-MYC-inducible bHLH-LZ transcription factor, which displays elevated expression in many types of tumors. We found that serum-starved *AP4*-deficient mouse embryo fibroblasts (MEFs) were unable to resume proliferation and showed a delayed S-phase entry after restimulation. Furthermore, they accumulated as tetraploid cells due to a cytokinesis defect. In addition, *AP4* was required for c-MYC-induced cell cycle re-entry. *AP4*-deficient MEFs displayed decreased expression of *CDK2 (cyclin-dependent kinase 2)*, which we characterized as a conserved and direct AP4 target. Activation of an AP4 estrogen receptor fusion protein (AP4-ER) enhanced proliferation of human diploid fibroblasts in a CDK2-dependent manner. However, in contrast to c-MYC-ER, AP4-ER activation was not sufficient to induce cell cycle re-entry or apoptosis in serum-starved MEFs. *AP4*-deficiency was accompanied by increased spontaneous and c-MYC-induced DNA damage in MEFs. Furthermore, c-MYC-induced apoptosis was decreased in *AP4*-deficient MEFs, suggesting that induction of apoptosis by c-MYC is linked to its ability to activate *AP4* and thereby cell cycle progression. Taken together, these results indicate that AP4 is a central mediator and coordinator of cell cycle progression in response to mitogenic signals and c-MYC activation. Therefore, inhibition of AP4 function may represent a therapeutic approach to block tumor cell proliferation.

## INTRODUCTION

The AP4 protein belongs to the group of basic-helix-loop-helix leucine zipper (bHLH-LZ) transcription factors [1]. AP4 exclusively forms homodimers, which bind to the E-box motif CAG/CCTG and either repress or activate the expression of target genes [2-7]. We previously identified the *AP4* gene as a direct transcriptional target of c-MYC and showed that the gene encoding the CDK-inhibitor *p21* is directly repressed by AP4 in human cells [8, 9]. Recently, we also characterized the regulation of *p21* by AP4 in mouse embryonic fibroblasts (MEFs) and showed that AP4 also controls the expression of the CDK-inhibitor *p16* in human and mouse cells [10]. In addition, we could demonstrate that deletion of *AP4* leads to premature senescence and that AP4 is required for cellular transformation by c-MYC and mutant RAS [10]. Moreover, we performed a genome-wide analysis of AP4-regulated genes and AP4 DNA binding in a human colon cancer (CRC) cell line [7]. Thereby we found that AP4 represents an epithelial-mesenchymal transition (EMT) inducing transcription factor (EMT-TF). We could further demonstrate that AP4 is crucial for metastases formation in a xenograft mouse model. Additionally, AP4 protein expression positively correlated with survival and distant metastases formation in the liver in two different colorectal cancer patient cohorts [7]. Moreover, elevated expression of AP4 correlated with poor patient survival also in gastric and hepatocellular cancer [11, 12]. Furthermore, we could identify a double-negative feedback-loop between AP4 and the tumor-suppressive microRNAs miR-15a/16-1, which controls the balance of EMT and mesenchymal-epithelial transition (MET) during metastasis [13]. Recently, it has been shown that AP4 is a target for proteasome-dependent degradation by the SCF/βTrCP ubiquitin ligase [14]. This degradation was shown to be mediated by phosphorylation of AP4 on a conserved degron. Interestingly, the ectopic expression of a stable AP4 mutant revealed that βTrCP-dependent degradation of AP4 is required for the fidelity of mitotic division.

The proto-oncogene *c-MYC* encodes a transcription factor of the bHLH-LZ class B, which binds to the E–box motif CACGTG (reviewed in [15]). c-*MYC* is commonly activated in human tumors via gene amplification, viral promoter insertion or chromosomal translocation, but also due to mutations of upstream regulators, such as APC/adenomatosis polyposis coli and β-catenin (reviewed in [16]). *c-MYC* is highly expressed in proliferating cells and becomes down-regulated when cells cease to proliferate. Deregulated *c–MYC* expression promotes cell proliferation and causes resistance to anti-mitogenic stimuli [17]. Constitutive *c-MYC* expression sensitizes cells towards apoptosis (reviewed in [18]).

The mechanisms which underlie the mitogenicity of *c-MYC* are only partially understood. It seems likely that the combined actions of multiple genes regulated by *c-MYC* contribute to the stimulatory effects of *c-MYC* on cell cycle re-entry and progression [19]. Several c-MYC-regulated genes encode components of the cell cycle machinery which control G_1_/S-progression, such as cyclin D1/D2 [20, 21], CDK4 [22] and CDC25A [23] or represent regulators of the G_2_/M progression, as e.g. MAD2 [24]. Accordingly, c-MYC also influences G_2_/M progression [24, 25].

In order to determine the function of AP4 during cell cycle progression, we analyzed *AP4*-deficient MEFs. Our results imply that AP4 is, at least in MEFs, a required mediator of cell cycle progression after mitogenic stimulation and c-MYC activation. This function of AP4 is at least in part mediated by direct induction of the central cell cycle regulator CDK2. Our analyses further revealed that AP4 function is required for successful completion of the final steps of cell divisions, as *AP4*-deficient cells display a cytokinesis defect resulting in tetraploid cells.

## RESULTS

### Role of AP4 in the mitogenic response

**Figure 1:**
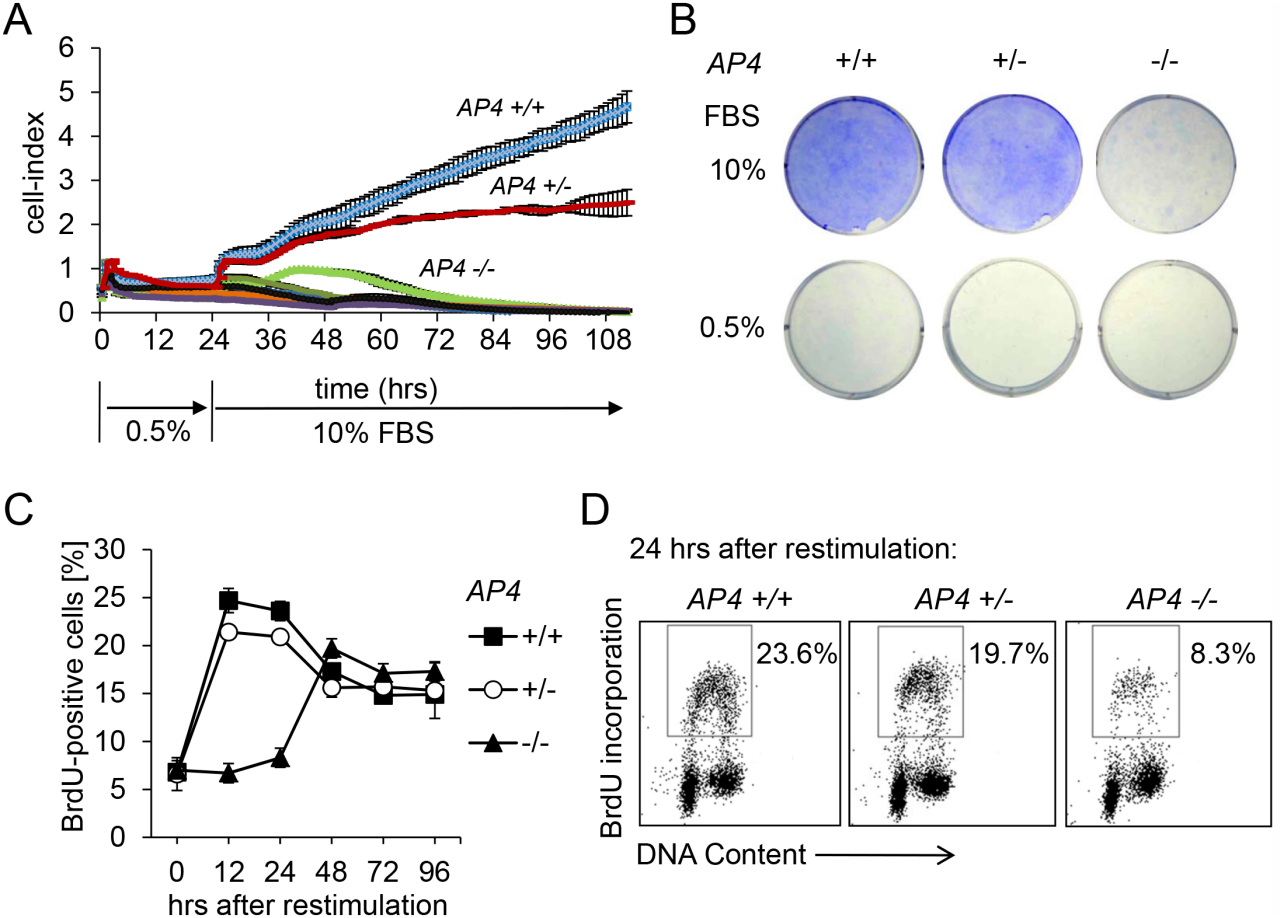
AP4 is required for proliferation after restimulation with serum **(A)** Cell proliferation analysis by impedance measurement. MEFs (passage 3) with the indicated genotypes were cultivated for 24 hours at 0.5% serum before release into medium containing 10% serum. Control cells were continuously kept in 0.5% serum. 2 × 10^3^ cells were seeded per well (96 well format) and analyzed over a period of 112 hours in one hour intervals. **(B)** 5 × 10^4^ MEFs (passage 3) with the indicated genotype were seeded per well into a six well plate and cultivated for 24 hours at 0.5% serum before release into medium containing 10% serum for 96 hours. Subsequently cells were fixed and stained with crystal violet. **(C)** Determination of DNA-synthesis after re-stimulation as described in A and B. BrdU was added for the last hour. At the indicated time points cells were subjected to flow-cytometric analysis. **(D)** Exemplary flow-cytometric results of the quantification depicted in (C) 24 hours after restimulation. Results in A and C represent the mean +/− SD (n=3).

In order to determine the function of AP4 during cell cycle progression we analyzed MEFs derived from *AP4* knock-out mice, which we had generated by deletion of *AP4* exons 2-4 [10]. The resulting mice with *AP4*+/− and *AP4*−/− germ-line deletion did not display any overt phenotype and gave rise to offspring with normal Mendelian ratios (Hermeking et al., manuscript in preparation). To generate MEFs, fibroblasts of embryos at day E13.5 were isolated and cultured as described previously [10]. In order to determine whether AP4 is involved in the proliferative response to mitogenic stimulation MEFs (passage 3) were kept at 0.5% serum for 24 hours and then re-stimulated by addition of medium with 10% serum (Figure 1A). *AP4*-deficient MEFs were unable to resume exponential proliferation in the following four days, whereas *AP4*+/+ MEFs immediately resumed exponential proliferation as determined by real-time impedance measurement. *AP4*+/− MEFs showed an intermediate proliferative response. These results were confirmed by crystal-violet staining of identically treated MEFs 96 hours after re-stimulation (Figure 1B). Re-stimulated *AP4*-deficient cells displayed a pronounced delay of S-phase entry as determined by BrdU-incorporation (Figure 1C and 1D). *AP4*+/− MEFs did not show a significant difference in the rate of BrdU-incorporation compared to *AP4*+/+ MEFs. As the DNA-synthesis was delayed but not completely abolished in *AP4*−/− MEFs the proliferation defect described above presumably is not due to an inability to enter S-phase. As described below *AP4*+/+ and *AP4*+/− MEFs display additional defects in cell cycle progression after mitogenic re-stimulation and an increase in double-strand DNA breaks, which may explain the inability to resume exponential proliferation. Taken together, *AP4*-deficient MEFs displayed a pronounced defect in their proliferative response after restimulation with serum which is presumably due to the deregulation of important cell cycle regulators in the absence of AP4.

### Characterization of *CDK2* as a direct AP4 target

**Figure 2:**
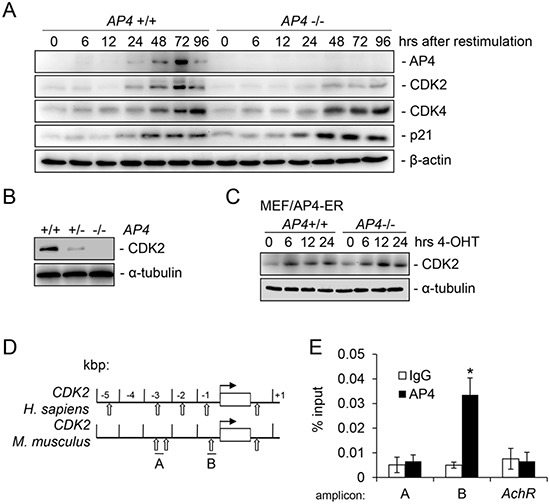
Characterization of murine *CDK2* as a direct target of AP4 **(A)** Immunoblot analysis of the respective proteins in MEFs (passage 3) with the indicated genotype harvested at the respective time-points after re-stimulation with 10% serum. β-actin served as loading control. **(B)** The expression of CDK2 was determined by immunoblot analysis in asynchronously growing MEFs (passage 3) of the indicated genotypes. α-tubulin served as loading control. **(C)** Immunoblot analysis of the indicated proteins in MEFs with indicated genotypes after AP4-ER activation by addition of 200 nM 4-OHT (4-hydroxy-tamoxifen) for the indicated periods. **(D)** Schematic representation of the genomic organization of the indicated human and murine promoters. Vertical arrows indicate AP4 binding motifs (CAGCTG). Horizontal bars indicate qChIP amplicons. **(E)** qChIP analysis of genomic DNA co-precipitated with an AP4-specific or mouse IgG control antibody in *AP4*+/+ MEFs. The mouse *acetylcholine receptor* (*AchR*) promoter lacking AP4 binding motifs served as a negative control. Results in D and F represent the mean +/− SD (n=3). Significance level as indicated: *p <0.05.

To identify factors involved in the proliferation defect caused by AP4 loss, we analyzed the expression of CDK2 as we recently identified *CDK2* as an AP4-target gene in a genome-wide screen for AP4-regulated genes in a human CRC cell line [7]. After re-stimulation, the induction of CDK2 was delayed and less pronounced in *AP4*−/− MEFs, whereas the induction of CDK4 was not affected by AP4 loss (Figure 2A). As reported previously, the induction of CDK4 by re-stimulation with serum is mediated by c-MYC [22]. Therefore, the observed differential regulation of AP4 targets is presumably not indirectly caused by the delayed cell cycle re-entry of *AP4*−/− MEFs. As expected, expression of p21 protein was increased in *AP4*-deficient MEFs. Furthermore, in asynchronous cycling MEFs the CDK2 protein level was decreased in *AP4*-deficient cells and showed intermediate expression in *AP4*+/− MEFs (Figure 2B). In addition, activation of a conditional AP4-ER (estrogen-receptor) fusion protein by addition of 4-OHT (4-hydroxy-tamoxifen) resulted in a rapid induction of CDK2 on the protein level in both, *AP4*+/+ and *AP4*−/− MEFs (Figure 2C). By chromatin immunoprecipitation (ChIP) and subsequent qPCR analyses we could detect AP4 occupancy at an E-box present 350 bp up-stream of the *CDK2* transcriptional start site (TSS) in MEFs (Figure 2D and 2E). Two other E-boxes located ~2.5 kbp upstream did not display AP4 occupancy. Therefore, the mouse *CDK2* gene represents a direct AP4 target gene.

### CDK2 is mediator of AP4-induced proliferation in HDFs

**Figure 3:**
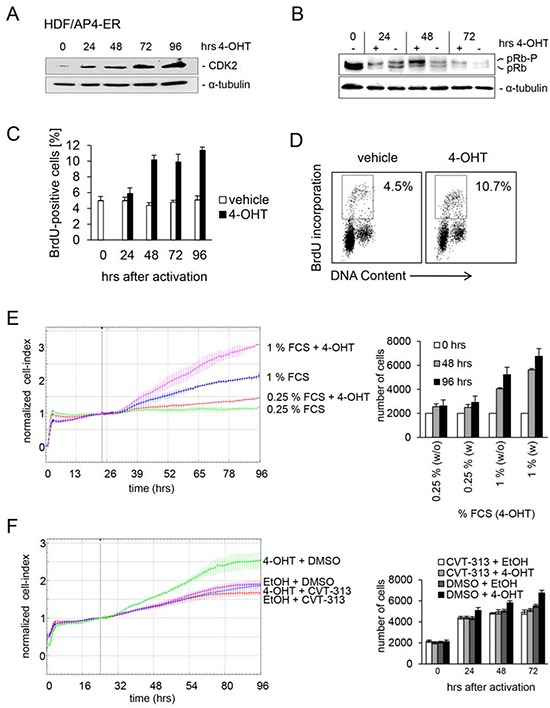
CDK2 is mediator of AP4-induced proliferation in HDFs An AP4-ER fusion protein consisting of AP4 and the estrogen receptor (ER) was stably introduced into human diploid fibroblasts by retroviral transduction. HDFs were kept in 1% serum for 24 hours. Then AP4-ER was activated by addition of 4-OHT. **(A)** CDK2 and α-tubulin protein expression was detected by immunoblotting after activation of AP4-ER for the indicated periods. **(B)** Detection of active, hypo-phosphorylated pRB and inactive, hyper-phosphorylated pRb-p protein by immunoblotting after activation of AP4-ER for the indicated periods. **(C)** Analysis of *de novo* DNA-synthesis after activation of AP4-ER by flow-cytometry. HDFs were kept in 1% serum for 24 hours before activation of AP4-ER by addition of 4-OHT (200 nM) for the indicated periods. Results represent the mean +/− SE (n=2). **(D)** Exemplary flow-cytometric results of the quantification depicted in (C) 96 hours after AP4-ER activation. **(E)** The respective cells were cultivated in the presence of the indicated serum concentrations for 24 hours before addition of 4-OHT. Cell proliferation was determined by impedance measurement (left panel) or by standard cell counting with Trypan blue exclusion (right panel). Results represent the mean +/− SD (n=3). **(F)** Effect of CDK2 inhibition on AP4-induced proliferation. The cells were kept in 1% serum for 24 hours and then treated with the indicated combinations of the specific CDK2-inhibitor CVT-313 (0.5 μM), inhibitor-vehicle (DMSO), 4-OHT (200 nM) and its vehicle (EtOH). Experiments in (C, E and F) were carried out in triplicates (n=3).

The activation of an AP4-ER fusion protein by addition of 4-OHT also resulted in increased CDK2 protein levels in human diploid fibroblasts (HDFs) in the presence of 1% serum (Figure 3A). This was accompanied by hyper-phosphorylation of pRB (Figure 3B). Since hyper-phosphorylated RB is an indicator for a cell cycle re-entry we analyzed the BrdU incorporation as a measure of DNA replication after AP4 activation. Indeed, AP4 activation at 1% serum led to enhanced DNA synthesis and proliferation, whereas AP4 activation was not sufficient to enhance proliferation at 0.25% serum (Figure 3C-E). Furthermore, treatment with the CDK2-inhibitor CVT313 [26] prevented the AP4-induced increase in proliferation at 1% serum (Figure 3F). These results show that CDK2 activation is required for AP4-mediated enhancement of proliferation in HDFs.

### *AP4*-deficiency results in a cytokinesis defect

**Figure 4:**
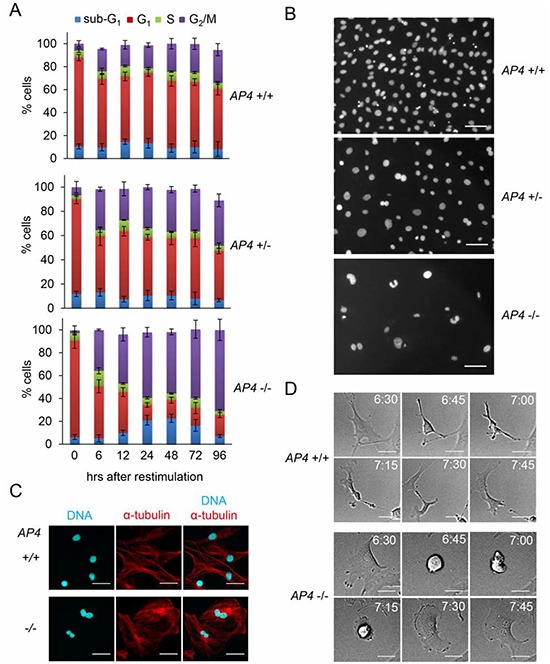
AP4-deficiency results in a cytokinesis defect **(A)** Determination of cell cycle distribution by DNA content analysis after release of serum-starved (0.5% FBS for 24 hours) MEFs (passage 3) into 10% serum containing medium. Per 25 cm^2^ flask 5 × 10^4^ MEFs with the indicated genotypes were analyzed. Results represent the mean +/− SD (n=3). **(B)** Nuclear DNA of MEFs (passage 3) treated as in (A) 96 hours after re-stimulationwas stained with DAPI and microscopy pictures were taken. The scale bar represents 10 μm. **(C)** Microscopic analysis of starved (0.5% FBS for 24 hours) MEFs (passage 3) with the indicated genotypes 96 hours after re-stimulation with 10% serum. Nuclear DNA was visualized by DAPI (cyan) and α-tubulin staining by indirect immunofluorescence (red). The scale bar represents 10 μm. **(D)** Representative picture sequences of MEFs recorded by time-lapse video-microscopy after re-stimulation with 10% serum. The time elapsed after re-stimulation is indicated in hours. The scale bar represents 10 μm.

The analysis of the cell cycle distribution of re-stimulated *AP4*-deficient MEFs revealed a substantial shift towards the tetraploid (4N) state indicative of a G_2_/M-arrest and a concomitant decrease of cells in the G_1_-phase (Figure 4A). We also observed an increase in apoptotic sub-G_1_ cells between 24 and 72 hours after restimulation of *AP4*-deficient MEFs. On the contrary, the majority of *AP4*+/+ MEFs were in the G_1_-phase 96 hours after re-stimulation. Microscopic examination of the DAPI-stained cells revealed that *AP4*−/− nuclei were generally enlarged and often showed invaginations and a butterfly-like shape (Figure 4B). The latter is indicative for a failed cytokinesis. These formations were seen at intermediate frequencies in *AP4*+/− nuclei, but not in *AP4*+/+ MEFs. Simultaneous detection of α-tubulin and nuclear DNA further confirmed that *AP4*-deficient MEFs often display two connected nuclei in one cell body (Figure 4C). By using time-lapse video-microscopy we could record incomplete cytokinetic events resulting in enlarged, binucleated cells in *AP4*−/− MEFs but not in *AP4*+/+ MEFs (see exemplary pictures in Figure 4D and Movie S1). Presumably, the cells resulting from such events are arrested in a G_1_-like state with a 4N DNA content. These results show that AP4 not only regulates the G_1_/S transition, but also has functions during and/or affects the G_2_/M transition.

### AP4 as a mediator of c-MYC function

**Figure 5:**
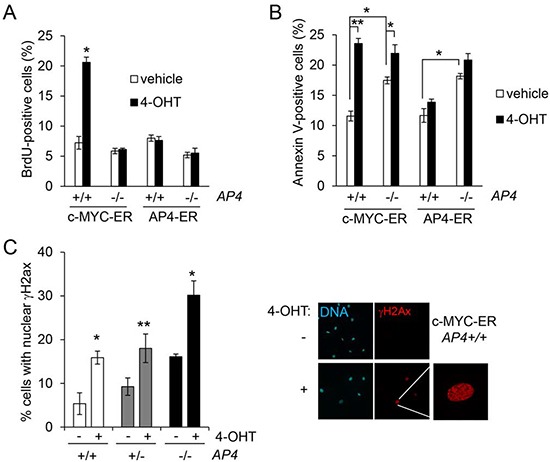
AP4 as a mediator of c-MYC function **(A)** Quantification of *de novo* DNA-synthesis by BrdU incorporation after c-MYC-ER or AP4-ER activation by addition of 200 nM 4-OHT for 24 hours. MEFs (passage 5) of the indicated genotype stably expressing virally transduced c-MYC-ER or AP4-ER were kept for 48 hours at 0.5% serum before the addition of 4-OHT. **(B)** Quantification of apoptotic cells by Annexin V staining, 48 hours after c-MYC-ER or AP4-ER activation. **(C)** Quantification of γH2Ax-positive nuclei by immunofluorescence 24 hours after c-MYC-ER activation in the indicated MEFs. γH2Ax was detected with a specific antibody and an Alexa-555-labeled secondary antibody, nuclei by DAPI staining. For every condition and genotype biological triplicates were analyzed microscopically with ten different fields for each replicate. Right panel, exemplary results of the described analyses. Results in (A-D) represent the mean +/− SD (n=3). Significance level as indicated: *p <0.05, **p <0.01.

We could previously demonstrate a direct regulation of *AP4* by c-MYC [8, 10]. Therefore, we determined the role of AP4 during c-MYC-induced cell proliferation and apoptosis in MEFs with *AP4*−/− and *AP4*+/+ genotypes. c-MYC-ER activation in serum-starved MEFs resulted in a more than three-fold increase in BrdU incorporation and therefore DNA-replication in *AP4*+/+ MEFs, whereas *AP4*−/− MEFs showed no significant increase in BrdU incorporation 24 hours after c-MYC activation (Figure 5A). AP4-ER activation in *AP4*+/+ or in *AP4*−/− MEFs did not results in a increase in DNA synthesis (Figure 5A). The relative increase in apoptosis after c-MYC-ER activation in serum starved MEFs was strongly decreased in *AP4*-deficient MEFs (Figure 5B): upon c-MYC activation apoptosis in *AP4*+/+ MEFs increased from ~12% to ~24% and therefore doubled, whereas *AP4*−/− MEFs only displayed a minor increase in apoptosis. Therefore, AP4 is required for c-MYC-induced DNA-synthesis and, at least in part, for c-MYC-induced apoptosis. These results furthermore support the notion that c-MYC-induced apoptosis is linked to c-MYC-induced cell cycle progression. In addition, ectopic AP4 expression is not sufficient, at least in serum-starved MEFs, for the induction of cell cycle re-entry and apoptosis.

Furthermore, *AP4*−/− MEFs showed increased levels of spontaneous apoptosis after serum starvation (Figure 5B; bars representing cells treated with vehicle). This phenotype might be due to the increased level of spontaneous DNA damage that we observed in *AP4*-deficient MEFs, as evidenced by an increase of nuclear foci of γH2Ax (Figure 5C), which represent sites of double-stranded DNA breaks and/or replication fork collapses [27]: whereas ~5% of serum-starved wild-type MEFs displayed nuclear γH2Ax signals, ~17% of the *AP4*-deficient MEFs were positive for γH2Ax. After c-MYC-ER activation a two-to-three fold increase of γH2Ax-positive nuclei was observed irrespective of the *AP4* genotype. However, the frequency of γH2Ax-positive nuclei reached ~30% in *AP4*−/− MEFs, whereas *AP4*+/+ MEFs showed ~16% γH2Ax-positive nuclei after c-MYC activation. Taken together, these results show that AP4 is necessary for an effective re-entry into the cell cycle after c-MYC activation. In the absence of *AP4*, the lack of AP4-mediated gene regulations may results in an increase in replication-related DNA damage, which interferes with the c-MYC-induced progression from G_1_- to S-phase. Therefore, AP4 is required for a coordinated cell cycle re-entry after c-MYC activation.

## DISCUSSION

By analysis of *AP4*-deficient MEFs we were able to identify new and important cellular functions of the bHLH-LZ transcription factor AP4. We found that AP4 is necessary for c-MYC-induced cell cycle re-entry and mitogen-induced cell cycle progression in MEFs, presumably by controlling the expression of the central cell cycle regulator CDK2. Our analyses also revealed that AP4 function is required during the G_2_/M transition, as *AP4*-deficient cells displayed a cytokinesis defect resulting in tetraploid cells.

Similar to the *AP4*-deficient MEFs analyzed here, *CDK2*−/− MEFs display a cell-cycle re-entry defect [28, 29]. Interestingly, *CDK4*-deficient MEFs also display a delayed entry into S-phase after serum re-stimulation [30, 31]. Presumably, AP4 regulates the activity of CDK4 by repression of the two CDK-inhibitors p16 and p21. Taken together, it is possible that the defects in S-phase re-entry and the premature senescence observed in *AP4*-negative cells are due to the decreased expression of CDK2 combined with elevated p21 and p16 levels, which inhibit the activity of the remaining CDK2/cyclin and other CDK/cyclin complexes.

The increase in spontaneous dsDNA breaks in *AP4*-deficient MEFs presumably results from replication stress caused by uncoordinated cell cycle events that may occur in the absence of *AP4*. The cytokinesis failure and subsequent accumulation of binucleated, tetraploid cells in *AP4*-deficient MEFs may be caused by aberrant events that occurred in the earlier phases of the cell cycle. For example, the elevated frequency of dsDNA breaks as evidenced by the increased number of γH2Ax foci in *AP4*-deficient cells can trigger pathways that regulate the expression of cytokinesis proteins, or their activity by post-translational modification and thereby block cytokinesis completion as discussed in [32]. The elevated expression of the two CDK inhibitors p16 and p21 in combination with the decreased expression of CDK2 might also contribute to the cytokinesis failure in *AP4*-deficient cells. For example, altered CDK activity was shown to affect centrosome duplication, which may result in mono- or multi-polar spindles, which ultimately may cause a defective cytokinesis [33-36].

In the future it will be important to further evaluate the role of AP4 in cell proliferation in models of c-MYC-induced tumorigenesis. In addition, the aberrant molecular processes underlying in the cell cycle defects of AP4-deficient cells that were described here deserve further analysis. Furthermore, our results suggest that inhibition of AP4 function, e.g. by small molecules blocking AP4 homodimerization, may be employed to therapeutically target c-MYC-driven tumor cells.

## EXPERIMENTAL PROCEDURES

### Generation of *AP4*-deficient mice

*AP4*-deficient mice and their phenotypes will be described elsewhere (Hermeking et al., in preparation). As described previously [10], the *loxP*-site flanked exons 2-4 of *AP4* were removed by crossing the respective mice with CMV-Cre mice. All ES cells and mice used had a C57Bl/6 background. The *Cre* allele was removed by further crossing. The respective genotypes of the mice and MEFs were confirmed by specific PCR analyses. Primers sequences for genotyping can be found in [10].

### Isolation and cultivation of mouse embryonic fibroblasts and human diploid fibroblasts

MEFs were isolated from day E13.5 embryos of the mice described above. The uterus was removed and washed in PBS. The yolk sacs were separated and the embryos were isolated carefully. The viscera of each embryo was removed and the embryo was washed twice in PBS, placed in trypsin-EDTA (Invitrogen), cut into smaller pieces and incubated for 10 minutes at 37°C. The cell suspension prepared from the embryo was washed with medium containing 10% FBS and plated in 10 cm culture dishes. MEFs and HDFs were routinely cultured in a humidified 5% CO_2_ and 20% O_2_ atmosphere at 37°C. MEFs and HDFs were maintained in Dulbecco's Modified Eagles Medium (DMEM, Invitrogen) containing 10% fetal bovine serum (FBS). MEFs were cultivated in presence of 100 units/ml penicillin and 0.1 mg/ml streptomycin. One passage represents treatment with trypsin and subsequent dilution of the cells at a ratio of 1:3 every three days. 4-hydroxy-tamoxifen (4-OHT, Sigma) was dissolved in ethanol (400 μM stock solution) and used at a final concentration of 200 nM. MEFs used in the same analysis were derived from littermates.

### Western blot analysis and antibodies

Cells were lysed in RIPA lysis buffer (50 mM Tris/HCl pH 8.0, 250 mM NaCl, 1% NP40, 0.5% (w/v) sodium deoxycholate, 0.1% sodium dodecylsulfate, complete mini protease inhibitors (Roche)). Lysates were sonicated and centrifuged at 16.060 x g for 15 minutes at 4°C. Per lane 30 – 80 μg of whole cell lysate was separated using 7.5% or 12% SDS-acrylamide gels and transferred on Immobilon PVDF membranes (Millipore). For immuno-detection membranes were incubated with antibodies specific for CDK2 (Santa Cruz), CDK4 (Santa Cruz), p21 (BD PharMingen), RB (BD PharMingen), β-actin (Sigma) or α-tubulin (Sigma). Signals from HRP (horse-radish-peroxidase)-coupled secondary antibodies were generated by enhanced chemiluminesence (Perkin Elmer Life Sciences, Boston, MA) and recorded with a CCD camera (440CF imaging system, Eastman Kodak Co., Rochester, NY).

### Retroviral infections

For retrovirus production and infection of MEFs Phoenix-E and for HDF cells Phoenix-A packaging cells were transfected with the respective pBabe vectors using calcium phosphate precipitation. Twenty-four hours after transfection, retrovirus-containing supernatants were harvested, passed through 0.45 μm filters (Millipore) and used to infect MEFs or HDFs in the presence of polybrene (8 μg/ml) four times in four hour intervals. Selection was started 48 hours later by addition of 2 μg/ml puromycin (Sigma) for five days.

### Phase-contrast microscopy

Images of cells in culture were captured using an Axiovert 25 microscope (Zeiss) with a Sony Digital Hyper HAD camera (Software: Kappa Image Base, Kappa Opto-electronics) or an Axiovert Observer Z.1 microscope connected to an AxioCam MRm camera with an Axiovision software (Zeiss).

### Plasmids

The generation of the plasmids used here was previously described in [10]. The pBabe-MYC-ER construct was kindly provided by Bruno Amati (Milan).

### Flow cytometric analysis of DNA synthesis and DNA content

To monitor DNA synthesis 50 μM 5-bromo-2’-deoxyuridine (BrdU, Roche) was added for 60 minutes at 37°C. Next, cells were harvested by addition of trypsin, resuspended and centrifuged at 300g for five minutes. After washing with phosphate buffered saline (PBS) cells were fixed by addition of ice-cold 70% ethanol and incubation for at least 30 minutes at -20°C. Fixed cells were resuspended in 0.1 mg/ml pepsin and DNA was denatured by incubation in 2 M HCl for 30 min at room temperature. After centrifugation (500g) cells were resuspended in 0.1 M Na_2_B_4_O_7_. Cells were washed once with PBS and PTS buffer (PBS, 0.5 % Tween 20, 2% FBS) respectively, and subsequently resuspended in 60 μl PTS + 6 μl anti-BrdU-FITC antibody (BD Biosciences Pharmingen) or an appropriate isotype control IgG and incubated for 30 min at room temperature in the dark. Next, cells were washed two times with PTS and resuspended in 500 μl PTS, 0.5 mg/ml RNase A (Sigma) and 50 μg/ml propidium iodide (Sigma). After incubation for 30 min at RT cells were analyzed by flow cytometry (CFlow6, Accuri). For DNA content analysis cells were harvested by addition of trypsin, washed and stained with a propidium iodide solution as described in [13]. Afterwards, cells were subjected to flow cytometry using a CFlow6 device (Accuri).

### Chromatin immunoprecipitation (ChIP) assay

Cross-linking was performed with formaldehyde (Merck) at a final concentration of 1% and terminated after five minutes by addition of glycine at a final concentration of 0.125 M. Cells were harvested with SDS buffer (50 mM Tris pH 8.1, 0.5% SDS, 100 mM NaCl, 5 mM EDTA) and resuspended in IP buffer after pelleting (2 parts of SDS buffer and 1 part Triton dilution buffer (100 mM Tris-HCl pH 8.6, 100 mM NaCl, 5 mM EDTA, pH 8.0, 0.2% NaN_3_, 5.0% Triton X-100)). Chromatin was sheered by sonication (HTU SONI 130, G. Heinemann) to generate DNA fragments with an average size of 500 bp. Preclearing and the incubation with AP4 antibody (AbD Serotec) or the respective IgG control (M-7023, Sigma) for 16 hours was performed as previously described [24]. Washing and reversal of cross-linking was performed as described [37]. Immunoprecipitated DNA was analyzed by qPCR and the enrichment was expressed as percentage of the input for each condition [37]. The sequences of oligonucleotides used as qChIP primers are listed in Table S1.

### Crystal violet staining

Cells were washed with PBS and fixed with 70% EtOH for 20 minutes at room temperature. Cells were stained with a crystal violet solution (5 mg/ml crystal violet (Sigma)) in 20% methanol for 30 minutes at room temperature. Then cells were washed extensively with water and the whole plate was photographed.

### Live cell imaging

After 24 hours of serum starvation with 0.5% serum MEFs were restimulated with a final concentration of 10% serum. 6 hours after restimulation the recording was initiated and performed for 42 hours in intervals of 15 minutes. For this an Axiovert Observer Z.1 microscope with an AxioCam MRm camera controlled by the Axiovision software (Zeiss) was used. Movies were recorded with a Plan-Apochromat 20x/0.8 M27 objective (Zeiss) at 37°C and 5% CO_2_.

### Assessment of proliferation by impedance measurements

The optimal cell concentration was determined by serial dilution for each cell line. For MEFs and HDFs 2 × 10^3^ cells were seeded per well (96-well). Subsequently, 100 μl of cell culture media at room temperature was added into each well of an E-plate 16 (Roche). Proper electrical-contacts and background impedance was measured. 100 μl cell suspension was added to medium containing wells on the E-plate 16. After 30 minutes incubation at room temperature the E-plate 16 was placed in a Xcelligence device (Roche) in a cell culture incubator. Impedance was monitored every 60 minutes for a period of up to 110 hours. The electrical impedance is represented as a dimension-less parameter termed cell-index (CI). All calculations were performed using the RTCA-integrated software of the Xcelligence system.

The unit-less parameter CI represents relative changes in electrical impedance caused by cells adhering to the surface of the wells which is calculated based on the following formula: CI = (*Z*i −*Z*0)/15, where *Z*i is the impedance at an individual point of time during the experiment and *Z*0 is the impedance at the start of the experiment. Impedance is measured at 3 different frequencies (10, 25 or 50 kHz) and a specific time (ref: Roche Diagnostics GmbH. Introduction of the RTCA DP Instrument. RTCA DP Instrument Operator‘s Manual, A. Acea Biosciences, Inc.; 2008.). To validate impedance measurements, cells were also seeded into 96 well plates in triplicates and counted at the indicated time points using a Neubauer-chamber.

### Apoptosis Detection with Annexin V

The protocol was performed according to the manufacturer's instructions (BD Pharmingen, 556570). Briefly, 8 × 10^4^ cells harboring c-MYC-ER or AP4-ER were seeded into a 25 cm^2^ flask. Cells were serum starved for 48 hours with 0.5% serum. For activation of c-MYC or AP4 4-OHT (200 nM) or the vehicle (ethanol) was added. After 48 hours cells were harvested by trypsination and washed twice with PBS. Cells were resuspended in 1 x binding-buffer (0.01 M Hepes/NaOH (pH 7.4), 0.14 M NaCl, 2.5 mM CaCl_2_) at a concentration of 1 × 10^6^ cells/ml. 100 μl of the solution (1 × 10^5^ cells) were incubated with 5 μl of FITC Annexin V and 5 μl propidium-iodid. Mixtures were gently vortexed and incubated for 15 minutes at room temperature in the dark. After the incubation 400 μl of the 1 x binding-buffer were added to each tube and the samples were analyzed within 1 hour by flow cytometry (CFlow6, Accuri).

### Immunofluorescence and confocal laser-scanning microscopy

For immunofluorescence analysis, cells were cultivated on glass cover-slides and fixed in 4% paraformaldehyde/PBS for 10 minutes, permeabilized with 0.2% Triton X 100 for 20 minutes and blocked in 100% FBS for 1 hour. Monoclonal mouse antibodies were used for detection of α-tubulin (DM 1A, T-9026, Sigma) and γH2AX (JBW301, Upstate) and Alexa Flour 555-conjugated anti-Mouse IgG was used (Invitrogen) as secondary antibody. Chromatin was stained by DAPI (Roth). Slides were covered with ProLong Gold antifade (Invitrogen). As a negative control all stainings were performed without primary antibody.

LSM (laser scanning microscopy) images were captured with a confocal microscope (LSM 700, Zeiss) using a Plan Apochromat 20x/0.8 M27 objective, ZEN 2009 software (Zeiss) and the following settings: image size 2048x2048 and 16 bit; pixel/dwell of 25.2 μs; pixel size 0.31 μm; laser power 2%; master gain 600-1000. After image capturing the original LSM files were converted into TIF files.

### Statistical analysis

Statistical significance was determined using unpaired two tailed Student's t tests.

## SUPPLEMENTARY FIGURES AND TABLES




