# Supplementary material for: AP4 is required for mitogen- and c-MYC-induced cell cycle progression
Source: Oncotarget. 2014 Aug 19;5(17):7316–27. doi: 10.18632/oncotarget.2348 (PMC4202125; doi:10.18632/oncotarget.2348)
Supplement: Supplementary file 1 [file oncotarget-05-7316-s001.pdf]

SUPPLEMENTARY FIGURES AND TABLES

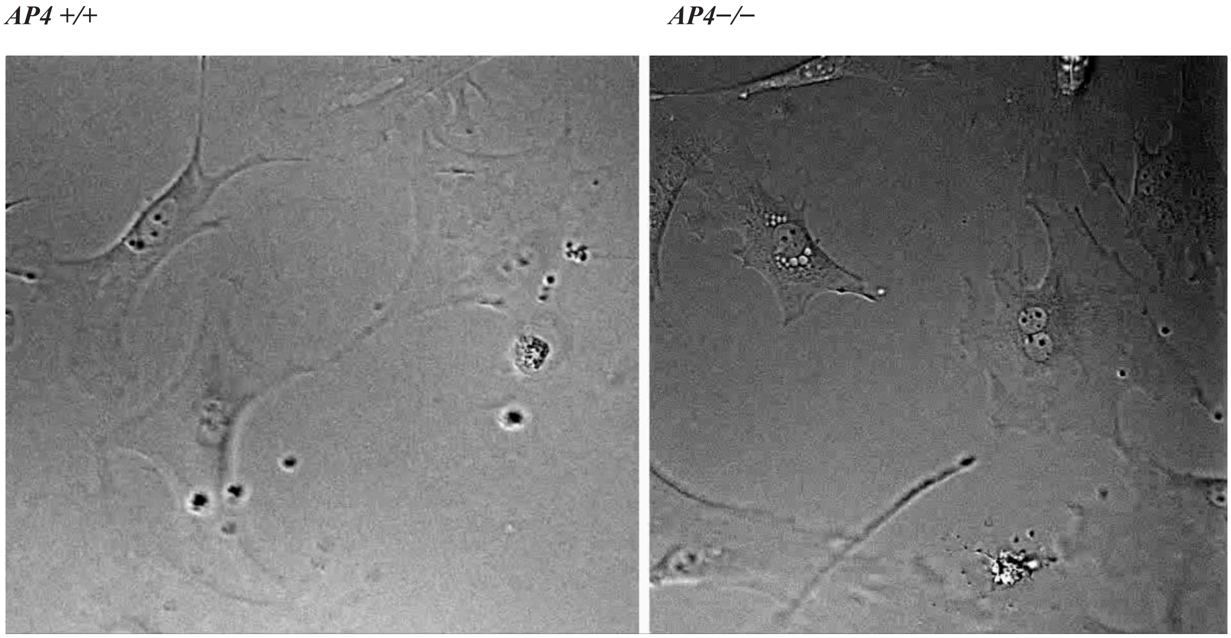

Supplementary Movie S1: Time-lapse video-microscopy of AP4 +/+ and AP4−/− MEFs. *AP4* +/+ MEFs (left window) and *AP4* −/− knock-out MEFs (right window) were starved for 24 hours in medium containing 0.5% serum and then released by addition of medium with a final concentration of 10% serum. 6 hours after restimulation the recording was initiated and continued for 42 hours. Pictures were taken in intervals of 15 minutes.

Supplementary Table S1. Oligonucleotides used for qChIP

Name	Sequence (5`-3`)
mouseAchR Fwd	AGTGCCCCCTGCTGTCAGT
mouseAchR Rev	CCCTTTCCTGGTGCCAAGA
mouseCDK2 -350 Fwd	ACGTGAACCAATCGGAAGG
mouseCDK2 -350 Rev	AGAGCTCAGCCCTTGACAAT
mouseCDK2 -350 Fwd	TGGGGGACAAAGCAGATATAA
mouseCDK2 -3500 Rev	CACCTGGCTCTTCTTCTTCG
